# Relation of Specialties to Each Other and to the Profession

**Published:** 1880-05

**Authors:** 


THE RELATION OF THE SPECIALTIES TO EACH OTHER, AND TO THE PROFESSION.—At page 204 of this Journal we attempted to say a few words that seemed to be called for, in order that certain Specialists might learn their interests would not be promoted by attending to general practice whilst advertising themselves devoted to a particular branch or specialty in medicine. And we desire to remark on this occasion, that subsequent reflection and observation intensify and strengthen what we then wrote on the subject of *Specialties and Specialists.* We have in our midst a number of gentlemen engaged in the practice of ophthalmic and aural surgery, gynæcological surgery, dental surgery, orthopedic surgery, the treatment of nervous diseases, skin diseases, throat and chest diseases, etc., etc., and we are happy to add, many of them are scrupulously careful not to trespass the boundary lines that separate them from general practice. The times in which we live render such division of professional labor both necessary and proper, and as no one of these can be esteemed more honorable, and probably not more important to the public, so the practitioner of any one of them cannot be more highly regarded than his brother specialist engaged in practicing any other one of these, all other things being equal; hence no one of these specialties should be esteemed more reputable as a calling than another. Whilst all the specialties are as "distinct as the billows, they are one as the sea" in the great art of healing disease and mitigation of human suffering and misery. We feel that *all* the various specialties are indeed "but parts of one stupendous whole," as we have occasion to deal with them in the pages of this Journal. Everything looking to the prolongation of human life and the melioration of human suffering will find cheerful greeting in these pages.

